# A Comprehensive Mini-Review on Lignin-Based Nanomaterials for Food Applications: Systemic Advancement and Future Trends

**DOI:** 10.3390/molecules28186470

**Published:** 2023-09-06

**Authors:** Ramachandran Chelliah, Shuai Wei, Selvakumar Vijayalakshmi, Kaliyan Barathikannan, Ghazala Sultan, Shucheng Liu, Deog-Hwan Oh

**Affiliations:** 1 College of Food Science and Technology, Guangdong Ocean University, Guangdong Provincial Key Laboratory of Aquatic Products Processing and Safety, Guangdong Province Engineering Laboratory for Marine Biological Products, Guangdong Provincial Engineering Technology Research Center of Seafood, Key Laboratory of Advanced Processing of Aquatic Product of Guangdong Higher Education Institution, Zhanjiang 524088, China; ramachandran865@gmail.com (R.C.); lsc771017@163.com (S.L.); 2 Department of Food Science and Biotechnology, College of Agriculture and Life Sciences, Kangwon National University, Chuncheon 24341, Republic of Korea; vijiselva10@gmail.com (S.V.); bkannanbio@gmail.com (K.B.); 3 Kangwon Institute of Inclusive Technology (KIIT), Kangwon National University, Chuncheon 24341, Republic of Korea; 4 Saveetha School of Engineering, SIMATS University, Kanchipuram 600124, India; 5 Collaborative Innovation Centre of Seafood Deep Processing, Dalian Polytechnic University, Dalian 116034, China; 6 Department of Computer Science, Faculty of Science, Aligarh Muslim University, Aligarh 202002, India; ghazala.sultan2k17@gmail.com

**Keywords:** lignin, nanomaterial, food applications, bioactive molecules, polyphenol

## Abstract

The shift to an environmentally friendly material economy requires renewable resource exploration. This shift may depend on lignin valorization. Lignin is an aromatic polymer that makes up one-third of total lingo-cellulosic biomass and is separated into large amounts for biofuel and paper manufacture. This renewable polymer is readily available at a very low cost as nearly all the lignin that is produced each year (90–100 million tons) is simply burned as a low-value fuel. Lignin offers potential qualities for many applications, and yet it is underutilized. This Perspective highlights lignin-based material prospects and problems in food packaging, antimicrobial, and agricultural applications. The first half will discuss the present and future studies on exploiting lignin as an addition to improve food packaging’s mechanical, gas, UV, bioactive molecules, polyphenols, and antioxidant qualities. Second, lignin’s antibacterial activity against bacteria, fungi, and viruses will be discussed. In conclusion, lignin agriculture will be discussed in the food industries.

## 1. Introduction

The world’s reliance on and overuse of fossil fuels have contributed to climate change, which has compelled researchers and businesses to concentrate their efforts on the investigation of renewable and environmentally friendly substitutes for coal, natural gas, and oil [1]. First-generation biological refineries resolve this problem by fermenting maize, sugar beets, and barley to produce bioethanol and trans-esterifying rapeseed and soybean oil to create biodiesel [2]. Even if these methods have been shown to be effective at producing green energy, the question of whether they can be considered sustainable remains unanswered. This is because they involve the usage of food crops and, as a result, compete with the production of food [2].

**Figure 1:**
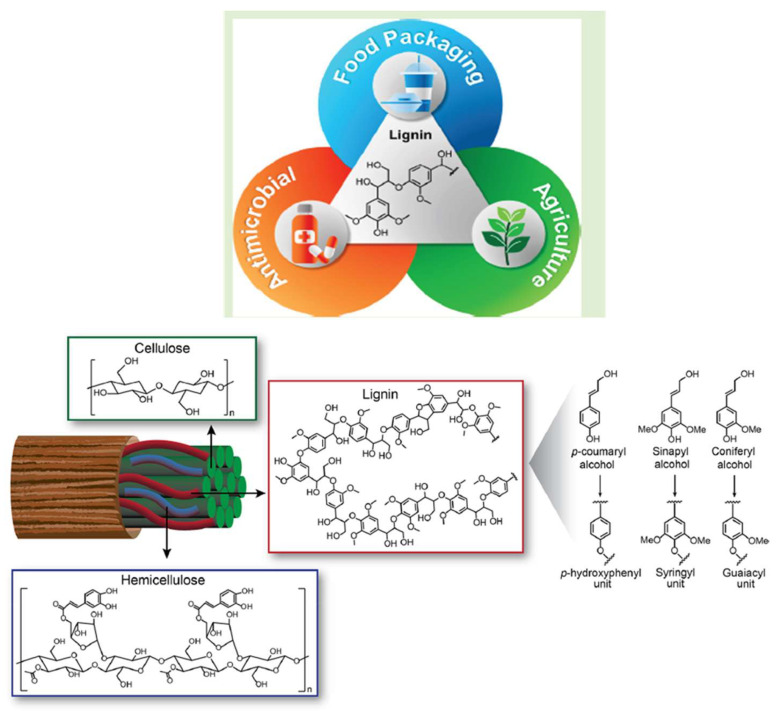
Representation of cellulose, hemicellulose, lignin, and lignin structural units.

A new generation of biorefineries is now being created, with the goal of making use of non-edible lignocellulosic biomass. This is being done to prevent issues such as deforestation, which is necessary to make room for the expansive acreage that these crops require, as well as a potential increase in the cost of food [3]. In particular, the valorization of lignin, which is one of the main parts of biomass, has a lot of potential to help bio-refineries grow in a way that is beneficial to society [4]. Lignin is a cross-linked aromatic hetero-polymer that, together with cellulose and hemicellulose, is a component of the plant cell wall within the plant cell wall; lignin is responsible for providing mechanical support as well as defense against pathogens. It is estimated that 15–35% of ligno-cellulosic biomass is composed of lignin, and approximately 100 million tons of this biopolymer are extracted annually as waste material from the paper and bioethanol industries [3]. Less than 2% of this large quantity is currently being put to use in the production of low-value goods, such as surfactants and adhesives, while the majority of the remainder is being burned up. Because of this, the use of this underutilized biopolymer as an application is appealing from the point of view of both the environment and the economy [5]. Laccases and peroxidases are two of the most important enzymes in the process of lignin biosynthesis. This process involves the oxidative radical polymerization of coniferyl, sinapyl, and p-coumaryl alcohol and is set in motion by a variety of enzymes. These structural units are referred to as guaiacyl, syringyl, and p-hydroxyphenyl units, respectively, once they have been incorporated into the lignin polymer [6]. Figure 1 illustrates the structure of lignin as well as the various building components of lignin, which is produced by coupling processes between phenolic radicals; the molecular weight distribution and composition of this biopolymer are quite heterogeneous. Furthermore, the molecular weight distribution and content of this biopolymer can vary greatly depending on the plant species [2].

Lignin can be extracted on an industrial scale from a wide variety of naturally occurring sources, including wood biomass, agricultural wastes, and energy crops. The extraction of lignin from biomass often involves one of these four primary bio-refinery processes: sulfite, soda, kraft [7]. In general, they use high temperatures and/or strongly acidic or basic conditions to cleave the lignin ether linkages, which results in the production of oligomers containing stable C–C bonds; because these bonds cannot be further changed, they prevent the lignin from depolymerizing into individual monomers. Several different research groups have come up with different strategies to prevent the formation of C–C bonds during the process of lignin extraction. Lignin can therefore be depolymerized, which results in the production of a significant number of aromatic monomers. As a result of this, the process of lignin valorization can include both the usage and application of the entire polymer, as well as the investigation of potential for the low-molecular-weight oligomers that are created through the process of lignin de-polymerization [8]. According to the findings of several publications, in addition to being beneficial to both the economy and the environment, lignin also possesses several inherent qualities that make it an appealing candidate for usage in a diverse range of applications [8]. We feel that food packaging, antimicrobial applications, and agriculture are three important and promising areas of application, and the purpose of this Perspective is to highlight opportunities and problems for the use of lignin-based materials in these three areas of application [9]. In this article, not only do we present an overview of the function that lignin plays in the three application domains, but we also highlight the obstacles and problems that still need to be solved, and we provide a forward-looking perspective on the probable future advances on this subject [3].

This review discusses current technologies and the potential for lignin valorization for food packaging, antimicrobials, and agricultural applications. Lignin has several applications; however, it might be difficult to use due to several issues. The structural and compositional variability of lignin depends on the plant source and extraction procedure and requires a detailed evaluation of both starting reagents and products. Due to its broad diversity, a well-defined structure–activity dependency should be created for most applications using this biopolymer. Lignin can be added to polymer films as a green additive to increase food packaging’s mechanical, gas barrier, antioxidant, and UV characteristics. Lignin’s compatibility with the matrix is a problem for polymer-film integration. To optimize compatibility and dispersion inside the polymer film, the lignin or nanoparticles must be functionalized to avoid heterogeneity and phase separation. Understanding the relationships between lignin and packaged items and film digestibility is also needed. Since lignin is barely degradable in composting settings, the effect of lignin incorporation on product composability and degradability should also be assessed. The mechanism of lignin’s interaction with bacteria, fungi, and viruses is uncertain and under controversy as an antimicrobial. Lignin’s molecular weight, impurity, and reactive group content variability allow many medical and biological applications but complicate its activity and safety assessment [10].

Despite several studies showing the antibacterial potential of lignin solution, only some have used it as a coating material for antimicrobial surfaces. Since viruses and bacteria can spread through polluted surfaces and systematic disinfection is laborious, microbe-inactivating coatings are important. Lignin coatings are easy to make and test against bacteria, fungi, and viruses. Additional studies should focus on coating preparation and performance against various diseases [11]. Agricultural applications and fertilizer use of lignin are explored in the last part of this Perspective. The existing fertilizer use could be more efficient. Lignin is a good starting material for controlled-release fertilizers, but significant difficulties must be solved for large-scale application. The preparation of lignin-based fertilizers needs improvement [12]. Lignin nanoparticles and lignin modified with nutrients other than nitrogen, such as phosphorus, are intriguing options. Another potential to boost crop output is lignin-based nano-fertilizers [12]. A viable approach is to build systems that release nutrients near the plant, reducing waste and environmental contamination. This Perspective highlights the potential of lignin, an underutilized natural source with great potential for food packaging, antibacterial, agricultural uses, and other technological difficulties requiring sustainable materials solutions [13,14].

## 2. Biosynthesis of Mono- and Oligo-Lignans and Their Function

Natural aromatic polymers of plant cell wall complexes, lignins are crucial to plant growth and development and have a significant impact on the efficiency with which sustainable lignocellulosic biomass may be applied. Formed primarily from three typical 4-hydroxyphenylpropanoids called monolignols (i.e., p-coumaryl, coniferyl, and cinnamyl alcohol), lignin polymers contain p-hydroxyphenyl (H), guaiacyl (G), and syringyl (S) units, respectively (Figure 1) [2]. There has been a lot of research into the monolignol biosynthetic pathway. Deamination of phenylalanine (or tyrosine) is the first step in the biosynthesis of monolignols, followed by subsequent aromatic hydroxylation, O-methylation, and simultaneous conversion of the side-chain carboxyl group to an alcohol group. In addition, several enzymes are involved in this biosynthetic route, including phenylalanine/tyrosine ammonia lyase and cinnamate dehydrogenase [15]. Caffeoyl shikimate esterase, caffeoyl-CoAO-methyltransferase, caffeoyl-CoA reductase, caffeic acidO-methyltransferase, and cinnamyl alcohol dehydrogenase are all enzymes involved in the production of cinnamyl alcohol [16]. After being generated and incorporated into an expanding lignin chain, the resulting monolignols are transported to the cell wall [16].

The relative amount of lignin components such as H, G, and S units and lignin structures varies among plant tissues, cell types, and developmental stages. Gymnosperm lignins typically have a high concentration of G units, arranged predominantly in an endwise way, and a low concentration of H units, which are common in compression wood zones. G and S units make up lignins in dicots, while H units are present in only minimal amounts; lignins in monocot grasses also contain G and S units, but at larger concentrations (although still 5%) [15]. It is important to keep in mind that the H-unit fraction is frequently overstated in grasses because the p-coumarate acylating lignin is wrongly quantified as H units. In addition, bioengineering allows for the manipulation of lignin composition (H/G/S), as evidenced by the dramatic enrichment of H units, which are a minor component of typical wild-type lignin, in HCT- and C3H-downregulated plants. In plants, lignin is made up primarily of G units when F5H is downregulated, and lignin is made up primarily of S units when F5H is overexpressed. Changes in content and structure caused by perturbations of genes in the lignin biosynthesis pathway are striking and suggest new methods for engineering lignin for improved applications [16].

## 3. The Biosynthesis of Lignin Formation

One of the requirements for living on land is a polymerization process called lignification, which results in lignin. Lignin monomers are transported to the secondary plant cell wall for oxidation and subsequent polymerization after production of monolignols. Lignin polymerization is a combinatorial radical coupling process, primarily in an endwise fashion, and it produces a series of substructures with a small variety of interunit linkages within the polymer, including b-*O*-4 (b-aryl ether), b-5 (phenylcoumarans), b-b (resinols), and 5-5 (biaryl, usually present in lignins as di-benzodioxocin units), b-1 (spirodienones), and 4-*O*-5 (diaryl ether) units [17]. Once radicals are formed, polymerization occurs through an enzymatic process that is independent of proteins. Electron delocalization around the aromatic ring and at the b-position of the side chain results in areas with single-electron density. This makes the phenolic radicals, produced by monolignol dehydrogenation, highly stable. The most common interunit linkages in the lignin polymer arise when these phenolic monolignol radicals undergo dimerization, leading to the formation of b-*O*-4, b-5, and b-dehydro-dimers [16]. The incoming monolignol radical exclusively undergoes b-*O*-4 coupling with the 4-*O*-phenolic function of the expanding oligomer to form b-*O*-4 alkyl aryl-ethers, which account for 50–80% of all interunit linkages in native lignin [18]. During the lignification process, oligomeric phenolic end units can couple with one another via 5-5- and 4-*O*-5-type connections, which do not originate from monomer–monomer or monomer–oligomer coupling. Furthermore, it has been demonstrated that b-1 originates from the interaction of a monolignol with a prepared b-aryl ether dimeric end unit. Because of the lack of regular and ordered repeating units, the lignin polymer generated by such a combinatorial radical coupling process is notoriously challenging to define and exploit. Lignins have been thought to be “highly branched” for quite some time [17]. Evidence for 5-5 (dibenzodioxocin) units and b-1 linkages, as well as the presence of free-phenolic 4-*O*-5 units in native gymnosperm lignin, which were previously thought to be branching points in lignin polymer, are increasing the clarity of lignin’s linear (non-branched) nature [19].

## 4. Potential Monolignols

Research on lignin biosynthesis has been increasingly important in recent years since lignin is the principal source of recalcitrance that hinders the efficient processing of lignocellulosic biomass [13]. As more and more lignin structural properties are revealed, the conventional understanding of lignin is extended, and a clearer picture of lignin production emerges. Numerous unorthodox units (other than H, G, and S units) have been identified as monolignols of lignin polymers; these include acetate, p-hydroxybenzoate (pBA), p-coumarate (pCA), monolignol ferulate conjugates, tricin, caffeyl alcohol, and 5-hydroxyconiferylalcohol, as well as the recently characterized benzoate (BA) conjugates [16]. The lignins of numerous plants, including kenaf and palms, contain up to 80% acetylation degree of native lignin, making hydroxy-cinnamyl acetates one of the most abundant unconventional monolignols. Willows, poplars, palms, and aspens are rich in hydroxy cinnamyl pBAs, while all grasses have hydroxycinnamoyl CAs [18]. Lignin is a sustainable source to obtain valuable products due to its abundance as a natural aromatic polymer. The existence of so many unusual monolignols suggests a high degree of lignification flexibility, a wide variety of lignin subunits, and a chemically complex lignin polymer, all of which expand the potential range of valuable products that can be extracted from it. Thanks to lignification’s adaptability, lignin structures can be engineered, considerably increasing the value of specific lignocellulosic materials [3,13].

## 5. Opportunities and Obstacles for the Lignin-Valorization Industry in the Food Sector

Most of the world’s lignocellulosic biomass is now regarded as low-value trash, despite being the most plentiful renewable resource on the planet. Lignin is an underappreciated bioresource because it is typically burned for energy production rather than transformed into anything of real value. The agro-food business generates copious amounts of trash that might be mined for high-quality lignin; thus, this is where research should focus. This study therefore offers a comprehensive synopsis of the development and progression of studies on lignin derived from the agro-food system (from 2012 to 2022) [15], covering both the extraction of lignin from various agro-food sources and its emerging applications in the agro-food chain. The highest-yielding crops on a yearly basis (*n* = 26) were chosen as possible lignin suppliers. Lignin purity and extraction efficiency (yield) were utilized to evaluate the raw material potential of the item. The overall interest in studying agro-food lignin as a source (567%) and an application (128%) has grown noticeably over the years [8].

To facilitate the shift toward a more environmentally friendly commodities economic performance, the research of renewable resources is crucial. Valorization of lignin can play an important role in this shift (Table 1). The aromatic polymer lignin makes up about a third of total lignocellulosic biomass and is extracted in enormous amounts as a byproduct of the biofuel and paper industries. There is a large supply of this renewable polymer at a low price since nearly all the 100 million tons generated annually are burned as poor-value fuel [2,6]. Regardless of lignin’s potential as a useful substance in a wide variety of contexts, its potential is still largely untapped. The purpose of this Perspective is to examine the potential and obstacles presented using lignin-based materials in antimicrobial, agricultural, and food packaging settings. Part one will cover current studies and potential future advancements in the application of lignin as an addition to enhance the mechanical, gas and UV barrier, and antioxidant capacities of packaging materials for food. To further elucidate lignin’s activity against bacteria, fungus, and viruses, we will then explore its potential utility as an antibacterial agent [8,10,13].

## 6. Lignin-Based Food Packaging

It is estimated that over 140 million tons of plastic are manufactured and used annually in the packaging industry. Approximately 40% of this is used for food packaging, the majority of which is not intended to be recycled and is designed for single consumption only. Food and food packaging make up almost half of all municipal solid garbage [22]. Polymers such as poly (ethylene terephthalate) (PET), polyethylene (PE), polypropylene (PP), polyvinyl chloride (PVC), and polystyrene (PS) are examples of some of the nonbiodegradable oil-derived materials that are most used for food packaging [22]. There are a number of different approaches that can be taken to lessen our reliance on resources that are derived from petroleum and to stop the buildup of waste products in the natural world. The installation of food storage devices that extend the amount of time that food can remain edible is one solution. Alternately, biodegradable polymers could be used as a stand-in for traditionally employed polymers in some applications [23] (Table 2).

**Figure 2:**
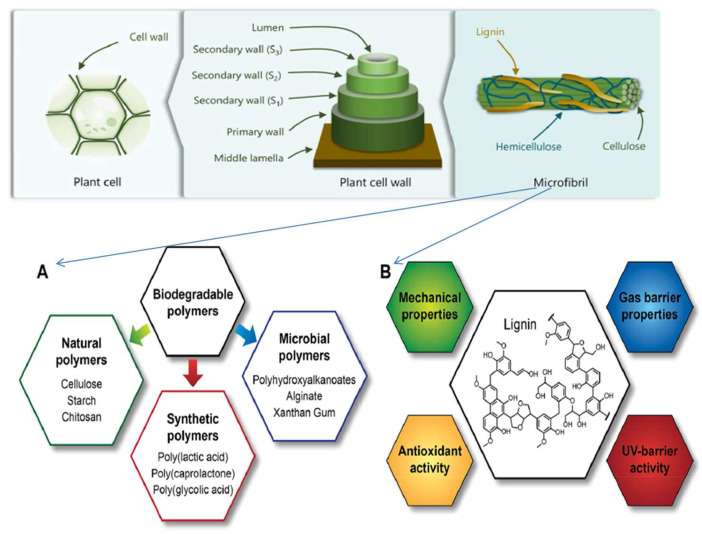
(**A**) Biodegradable polymers used in food packaging. (**B**) Properties that lignin incorporation can affect, when incorporated in a biodegradable polymer film.

Microorganisms can break down biodegradable polymers into CH_4_, CO_2_, and H_2_O. Based on where they come from, biodegradable polymers can be categorized as natural, microbial, or manmade [26]. It is essential to emphasize that the end-of-life management of these materials, which may include commercial composting or composting at home for some of them, continues to be just as vital as it was in the case of the oil-derived products. Even though polymers can be used to replace plastics that do not break down and make less of an impact on the environment, they do not have as good mechanical and barrier qualities and are often more expensive than widely used materials [21]. For these reasons, they make up only 1% of the plastics used for food packing. One approach that can be taken in the direction of accomplishing this objective is the utilization of biodegradable polymers that contain fillers made from lignin. Incorporating lignin into food packaging films has the potential to affect the films’ mechanical and gas barrier qualities. Additionally, the insertion of lignin can give the packaging material with properties such as antioxidant and UV barrier activities [32] (Figure 2).

Lignin can be introduced into a polymer in one of two ways: either by mixing free lignin with the polymer of interest, or by employing lignin nanoparticles instead. Several case studies illustrate the usage of biodegradable polymer films containing lignin as filler [32]. This biopolymer can be used not only for the mixing of free lignin but also for the incorporation of nanoparticles into food packaging films. The synthesis of lignin nanoparticles can be accomplished by a variety of approaches, including precipitation using a solvent or pH exchange, self-assembly, microwave assistance, ultra-sonication, and aerosol processing [31,32]. To make nano-composites, these particles can be mixed into a matrix in the appropriate proportions. The fact that nanoparticles have a very high surface area relative to their volume is the primary benefit of employing them. There are some examples of lignin nanoparticles being incorporated into a biodegradable polymer matrix to generate nano-composites. These nano-composites have the potential to be used for food packaging [33].

Nanoparticles tend to group together into greater clusters as time goes on. This is because the aromatic cross-linked lignin and the polymer matrix do not get along very well. This challenge can be conquered by modifying the surface of the lignin nanoparticles to make them more compatible with the matrix that is covered [27]. Citric acid was used in one experiment to etherify the surface of lignin nanoparticles, which was one method for accomplishing this goal [34]. It is possible to integrate 10% of lignin nanoparticles into a PVA film, and grafting polymer chains onto the surface of the particle is yet another method [35]; for instance, ring-opening polymerization of lactide was used to make PLA-modified lignin nanoparticles [35]. These nanoparticles could be included in a well-dispersed manner to generate PLA films that contained up to 10% lignin nanoparticles [36]. Ring-opening polymerization of lactide was also used to generate PLA films. As was indicated earlier, the incorporation of lignin into food packaging materials makes it possible to improve their mechanical and gas barrier qualities, in addition to their antioxidant activity and UV barrier capabilities. This makes the incorporation of lignin an appealing strategy [37]. It is possible for the mechanical characteristics of a polymer film to shift in a variety of ways once lignin has been added into the film. The tensile strength and elongation at break can increase, decrease, or stay the same depending on the type of lignin and the polymer matrix [35]. The effect of lignin incorporation is film-specific; however, the main takeaway from the research is that the compatibility between the lignin filler and the polymer matrix is what ultimately determines the mechanical properties of the film [38].

In general, increased lignin dispersion and compatibilization efficiency leads to improvements in the material’s mechanical properties. Lignin esterification, the use of cross-linkers, and polymer surface modification are all potential strategies that might be implemented to prevent phase separation and improve the compatibility of lignin with the polymer matrix [35]. Incorporating lignin can make the film less permeable to oxygen and water vapor, which is especially beneficial in situations when the film is formed from hydrophilic elements like alginate and starch [34]. This is not only due to the hydrophobic property of lignin, but also due to the interaction of lignin with the film matrix. Lignin, once it has been integrated into the film, interacts with the hydrophilic groups of the biopolymer, lowering the biopolymer’s attraction for water and oxygen molecules because of these interactions. The oxidation of lipids and proteins within food is one of the primary causes of food spoilage. This process influences the appearance, flavor, and odor of the food, and it can also result in the production of poisonous aldehydes. It is possible to avoid the oxidation of food by incorporating antioxidant chemicals into the materials used for food packaging [35]. These compounds work as radical scavengers and delay the processes that are caused by radicals. Butylated hydroxyanisole and butylated hydroxytoluene are two examples of the types of antioxidants that are commonly used. Although they are particularly effective in preventing the oxidation of food, the production of these compounds might result in the formation of allergenic substances such as sulfites, nitrates, and benzoic acid [35,36]. These substances can also have additional negative effects on human health. As a result, there has been a recent uptick in people looking for natural antioxidants that are friendlier to the environment and safer [38].

Lignin is an effective antioxidant that shows promise as a potential replacement for the synthetic chemicals listed before. The presence of phenols in the structure of lignin, which can act as radical scavengers, is the reason for lignin’s ability to possess antioxidant properties [35,38]. A higher level of antioxidant activity in lignin has been shown to correspond with a lower molecular weight and smaller discrepancies, as well as higher phenol content. Several investigations have corroborated this correlation. This polymer can absorb light in the ultraviolet range (200–400 nm) as a result of the presence of chromophores inside its structure. Some examples of these chromophores are carbonyl and conjugated phenol groups [39].

Lignin fillers offer several benefits for the food packaging industry, one of which is the ability to shield food from the damaging effects of ultraviolet (UV) radiation. Because of the brown hue of the lignin, it is essential to keep in mind that the polymer film will lose some of its visual transparency because of the UV protection that is provided by the lignin [40]. Customers typically want to be able to see the actual product they are purchasing through the container, making visible transparency an essential component of food packaging [40]. Therefore, it is always vital to optimize the lignin concentration and distribution inside the polymer film to establish a material composition in which the film protects the food from UV irradiation while simultaneously allowing the product to be visually visible. The use of lignin in food packaging applications faces several challenges but also has promising prospects for the future. It is possible to improve the performance of biodegradable polymer films used for food packaging by including lignin into those films [41].

Lignin can be added to biodegradable polymers to improve their mechanical and gas barrier qualities, which are two of the primary shortcomings of biodegradable polymers [40]. Lignin can also give biodegradable polymers antioxidant and UV barrier capabilities, which are extremely important for the preservation of food. The creation of such mixed materials has several issues, one of which is ensuring that the polymer matrix, which is typically composed of linear aliphatic polymer chains, and the aromatic cross-linked structure of lignin are compatible with one another. The film develops phase separation and heterogeneity, both of which reduce the performance of the product. The same principle applies to the manufacturing of nano-composites, which involves embedding lignin nanoparticles in a polymer film. Aggregation and phase separation are required for larger particle content; only very modest amounts of nanoparticles have been incorporated into these films [40,41]. Higher particle content is not possible without these two processes. In order to obtain an effective dispersion of lignin in the final film, it is essential to functionalize lignin and lignin nanoparticles in order to increase their affinity with the polymer matrix. Regarding the utilization of lignin in food packaging, the matter of safety is another significant concern that has yet to be addressed and needs to be. The research that has been done thus far into how lignin interacts with packaged food and how it is digested in living organisms is, at this point, only extremely preliminary and will require further inquiry [42].

The design of environmentally friendly food packaging must consider how the product will be disposed of once it has reached the end of its useful life. Most of the materials that make up food packaging are thrown away in landfills rather than being recycled. This is due to the inclusion of additives, as well as food contamination, which can be difficult to separate. Composting is a viable alternative treatment option for end-of-life waste, in contrast to landfilling, which results in the occupation of significant quantities of area as well as the emission of greenhouse gases [43]. According to the American Society for Testing and Materials (ASTM), a plastic is considered compostable if it “undergoes degradation by biological processes during composting to yield carbon dioxide, water, inorganic compounds, and biomass at a rate consistent with other known compostable materials and that leaves no visible, distinguishable, or toxic residue” [44]. This definition applies to plastics that degrade into nontoxic byproducts during the composting process. This is a subgroup of biodegradable plastics, which are described as “plastics that break down because of the action of naturally occurring microorganisms like bacteria, fungi, and algae.” As a result, not all compostable plastics are made from biodegradable plastics [45].

Lignin is efficiently biodegraded by white-rot fungi and numerous types of bacteria, but the degradation of lignin under decomposition conditions, which are typically used to dispose of food packaging, is incomplete and inefficient. Additionally, the qualities introduced by the addition of lignin in a polymer matrix, such as improved gas barrier, decreased water permeability, and increased hydrophobicity, can lower the material’s degradability in the conditions of composting [46]. These properties include improved gas barrier, decreased water permeability, and increased hydrophobicity. Since this variable is frequently overlooked in previously conducted research, it is imperative that further investigation be directed toward determining how the incorporation of lignin affects the end product’s composability [47].

## 7. Food Packaging Barrier Concepts

Taste, smell, longevity, and marketability of food goods are greatly affected by packaging. Due to packaging issues, 40% of food worldwide degrades each year. Barrier qualities protect food inside a packaging by preventing moisture (water vapors), oxygen, carbon dioxide, grease, and oils from entering/exiting. Packaging must resist water, oxygen, grease, microorganisms, aromatic chemicals, carbon dioxide, and oils [48]. Paper/cardboard packaging is permeable to gases, water vapors, and liquids. Instead, plastic-based packaging has a wide mass transfer characteristic with low to good barrier value, which is crucial for food packaging. Synthetic polymers including PE, PP, and EVOH have been widely employed to improve paper-based packaging barrier properties [49], such as coffee cups and cotton board burger boxes, where synthetic polymers laminate the inside. However, employing petroleum-based polymers in cardboard board/paper packaging produces environmental and economic problems. Since polymers are not water-soluble, recycling cardboard packaging with an inner plastic lamination is difficult [48]. This makes recycling less profitable and disrupts the circular economy. To address this issue, researchers from several professions and sectors are seeking a bio-based alternative to petroleum-based paperboard laminations.

The barrier qualities of a polymeric packaging material determine its product protection capability and product specification and end-use applications substantially affect barrier characteristics such as thickness, crystallinity, size, and permeant polarity, which affect the barrier qualities of packaging materials like film and paperboard [49]. Permeants are affected by humidity and temperature during storage, and the cohesive energies between the barrier wall and molecule determine permeant entry and exit; greater permeation results in the lowest cohesive energies between the barrier wall and molecules, while non-permeant molecules have greater cohesive energies [50]. Water vapor and oxygen are major permeants because they can cross the polymeric wall and impair food quality. Glass, metal, and plastic are used to package dairy products, fresh produce, meat, ready-to-eat food, juices, and water. These materials are used because they block water and other permeants; however, the widespread use of conventional packaging materials raises transportation and recycling expenses; furthermore, they can pose environmental issues because they do not decompose in landfills [50]. Bio-based plastics are a possible alternative to petroleum-based ones. Biopolymers like PLA, cellulose, chitosan, alginate, starch, zein, whey protein, PHA, and others have been tested in food packaging for decades [51]. Considering the current literature, research has focused on improving these biopolymers’ barrier properties to compete with petroleum-based polymers like PET, EVOH, PVDC, nylon, and polycarbonate [52]. A water barrier is a key barrier property connected to the material’s end-use application, and the highly hydrophobic surface is needed to preserve packaging for liquid beverages with high water content. Milk products are packaged with a PET (very hydrophobic > 150°) water barrier, aluminum gas and UV-light barrier, and carton boards for structure and printability [40,41]. Cellulose, starch, and alginate are bio-based polymers that could replace PE lining. Their greater hydrophilicity is the key issue before employing such biopolymers, and the OH groups on the backbone of polysaccharide-based polymers (cellulose, chitosan, starch, etc.) make them hydrophilic [51].

## 8. Evaluation of Lignin-Based Biopolymer Recyclability

Plastic is now used in numerous products and ubiquitously. Food packaging, construction, garments, adhesive, insulation, and other uses employ plastics; however, the plastic sector faces many difficulties today. Pollution and sustainability issues remain unresolved [23]. Most plastics on the market are made from nonrenewable fossil feedstock, making our lifestyle unsustainable as traditional plastics are hard to recycle and do not decay naturally [46]. Recently, bio-based polymers derived from sources like sugarcane, corn, microalgae, and sugar beets have been explored. With growing environmental awareness, there’s an increasing consumer demand for sustainable plastics. This has piqued the interest of both research and industry sectors in creating plastics from non-oil-based materials [46]. Currently, bio-based plastics hold less than 1% of the market share, being overshadowed by conventional plastics primarily due to their higher cost compared to fossil-derived feedstocks [23]. However, as with any emerging technology, costs are expected to decrease with further research and development. Critics suggest that, in the future, biodegradable plastics could even find use in food production [46]. A prevailing challenge for society is to diminish the use of long-lasting, non-biodegradable packaging. Sustainability in production and consumption remains a significant driving force across various sectors, especially in the packaging industry. This trend is evident as more companies are introducing recyclable, bioplastic, and paper-based packaging options [46,51]. The goal is to provide nontoxic, sustainable, biodegradable, low-cost materials with good physical appearance, barrier qualities, and thermo-mechanical stability. Lignin would not harm the environment after its service life due to its material characteristics, biodegradability, and non-phytotoxicity [26,27].

The extracted lignin from wood chips waste (obtained from the paper industry) can be blended this with starch and gelatin to produce lignin-starch and lignin-gelatin biodegradable polymeric films [46]. There are several reports focusing on the potential use of lignin in combination with their biopolymers (e.g., blending of starch/gelatin with lignin) and with chemical modifications (for polypropylene films) to produce packaging films for wider food industry applications [46]. Lignin being a natural component of fiber, a strong bonding exists between the lignin matrix and the fiber component. Natural composite films produced from this combination are always strong and display good mechanical properties [42]. Furthermore, food packaging films developed from fiber/lignin-based materials can always be a good replacement for oil/petroleum-based polymers. Lignin used in different forms as a filler material (e.g., organosolv, kraft, soda lignin) is reported to exhibit better mechanical properties [49,50]. The electro-spinning of C-lignin (devoid of chemical treatments obtained from vanilla seeds) into fibers was successful and is foreseen to find wide industrial applications [52]. Lignin-based hydrogels are gaining popularity owing to superior water absorption and retention properties, tissue imitating abilities, low toxicity, and biocompatibility as well as controlled diffusion of desirable materials [28,30].

## 9. The Role of Lignin as an Antimicrobial Agent in Food Preservation

Some bioactive substances that can be derived from plants can be employed as antimicrobial agents to stop the growth of pathogenic bacteria, fungi, and viruses. Examples of such compounds include polyphenols, amino acids, terpenoids, flavonoids, and tannins [53]. These compounds are fascinating not only because of the biological activity they exhibit, but also because of their biocompatibility, renewability, and biodegradability. Isolation of most of these compounds, however, is notoriously difficult due to the extremely low concentrations at which they are normally found in plants and the labor-intensive extraction techniques that are typically required. In recent years, lignin has come to garner a lot of attention since it is readily available, does not cost much, and demonstrates some fascinating biological functions [46]. The inherent ability of lignin to shield plants from the attack of pathogens is the source of this compound’s antibacterial effect. Lignin could stop bacteria and fungus from degrading carbohydrates by acting as a barrier between them and the plant’s internal environment. Antibacterial, antifungal, and antiviral properties have been seen in technical lignins that have been isolated from lignocellulosic biomass [52] (Table 3). These observations have been made in the context of biological and medical research. It is generally agreed that the phenolic hydroxyl groups in lignin are responsible for the antibacterial activity of lignin. These groups can cause damage to the cell membrane of bacteria, which ultimately results in the lysis of the bacteria [53]. It is common knowledge that phenols and polyphenols possess antibacterial properties; however, the specific mechanism of action behind this property is not yet fully understood. Both the type of lignin and the strain of bacteria play a role in determining the level of antibacterial activity produced by the compound. The kraft lignin that was extracted from maize was able to effectively deactivate Gram-positive bacteria such as *Staphylococcus aureus* and *Listeria monocytogenes*, but it did not have the same effect on Gram-negative bacteria or bacteriophages. Inactivating Gram-positive bacteria like Bacillus cereus, *Staphylococcus aureus*, and *Pseudomonas aeruginosa*, as well as Gram-negative bacteria like *Escherichia coli* and *Salmonella enteritidis*, is possible with kraft lignin that is produced from eucalyptus [54]. Lignin can be employed in its purest form as an antibacterial agent, but it can also be combined with other antibacterial agents or incorporated into more complicated systems. Lignin is wrapped around a core of silver nanoparticles to obtain exceptional antibacterial activity against *Staphylococcus aureus* and *Escherichia coli* without the formation of silver ions that are harmful to the environment [55]. The antibacterial activity of polymer films including lignin as filler has also been investigated in a few studies. These polymer films were made in a manner identical to the polymer blends mentioned in the previous paragraph, which demonstrated successful inactivation of a variety of infectious agents [56].

The antiviral properties of lignin–carbohydrate complex and lignosulfonate, both of which are soluble in water, have been examined against a variety of viruses in cell culture media as well as in aqueous solution [57]. Both compounds are water-soluble. Although some research organizations have attempted to establish a link between the molecular makeup of lignin and the antiviral impact, the well-defined antiviral mechanism has not yet been elucidated [57]. Lignin–carbohydrate complex is formed when lignin is covalently bonded to carbohydrates inside the plant wall. This complex can be removed from biomass using a variety of processes, including acidolysis, fractionation, and enzymatic hydrolysis. Inactivation of the encephalomyocarditis virus (EMV) and the herpes simplex virus (HSV) has been demonstrated to be accomplished by lignin–carbohydrate complexes [57]. Their antiviral activity in aqueous solution was linked to the suppression of viral binding and penetration into the host cells. This was found to be the case. It is not yet known what function lignin specifically plays in the antiviral action of the lignin–carbohydrate complex [58]. Both the herpes simplex virus (HSV) and the human immunodeficiency virus (HIV) have been demonstrated to be susceptible to the antiviral properties of lignosulfonate, which is the only kind of technical lignin that is water-soluble. It was determined that the antiviral action of lignosulfonates was due to their structural similarities with heparan sulfate. Heparan sulfate is a proteoglycan that can be found in close proximity to the cell wall. This is an area where viruses can typically interact with cells [58]. The antiviral strategy has not been completely elucidated; nevertheless, it has been demonstrated that it is affected by the amount of sulfur present, the molecular weight, and the counterion (Na^+^, Ca^2+^, NH_4_^+^) [59].

**Table 3:** Lignin as antimicrobial agent in solution.

Lignin Type	Concentration (mg/mL)	Inactivated Pathogen	Reference
DMSO Medium			
Kraft lignin	15	*Escherichia coli*, *Staphylococcus aureus*, *Pseudomonas aeruginosa*, *Salmonella enteritidis*, *Bacillus cereus*	[60]
Pyrolytic lignin	5	*Staphylococcus aureus*, *Escherichia coli*	[61]
Organosolv/Kraft lignin	1–20	*Aspergillus niger*	[62]
Organosolv lignin	0.48–0.025	*Candida parapsilosis*, *Candida krusei*, *Candida guilliermondii*, *Candida albicans*,	[63]
	0.5, 5, 10	*Aspergillus niger*, *Saccharomyces cerevisiae*	[64]
Cell Culture Medium			
Lignosulfonate	70 nM–236.6 μM	HIV	[65]
	0–0.2	HIV	[66]
	0–0.5	HIV, HSV	[67]
Others			
Kraft lignin	100	*Listeria monocytogenes*, *Staphylococcus aureus*	[68]
Lignin-	0.05	Encephalomyocarditis virus (EMV)	[69]
carbohydrate	0.5	Herpes simplex virus (HSV)	[70]
complex	0.1, 2	EMV, HSV	[71]
Ligno-sulfonate	10	Human immunodeficiency virus (HIV), HSV	[72]

## 10. Challenges and Future Perspectives for the Application of Lignin as Antimicrobial Agent

It has been demonstrated that lignin is an effective agent for inhibiting the growth of bacterial, fungal, and viral pathogens. The heterogeneity of lignin in terms of its structure, the number of reactive groups it has, and the impurities it contains is the primary obstacle for using lignin as an antibacterial agent [67]. It is possible to acquire lignin from a wide variety of natural sources and by employing a wide variety of techniques; as a result, lignin’s characteristics and its effectiveness against pathogens can be extremely variable; therefore, to develop a structure–activity dependency profile, one must first have a fundamental mechanistic understanding of how lignin inhibits the activity of microorganisms including bacteria, fungi, and viruses [68]. Although certain fungi, bacteria, and enzymes can break down lignin in the environment, it is yet unknown what will happen to this polymer inside of a human body [69]. In addition, despite the numerous research that have been conducted on the biocompatibility of lignin, the effects that the utilization of lignin for the purposes of biomedicine would have on cells and genes are still mostly unclear and will require further exploration [73] (Table 4).

Several phenol monomers identified and extracted from lignin that demonstrated effective antiviral activity against the encephalomyocarditis virus are noteworthy to mention in relation to the studies about viral inactivation, and this suggests that phenolic groups are involved in the antiviral activity of lignin in addition to its use as a macromolecule [54]. For the purpose of this application, only water-soluble lignosulfonates and solution-based lignin carbohydrate complexes have been evaluated for efficacy. In a recent study, antiviral lignin surface coatings made of lignins that are not soluble in water were created. These coatings effectively inactivated HSV-2 (>99% after 30 min). The mechanism that lies behind the antiviral activity of these coatings has received a significant amount of research, and it was discovered that this mechanism is highly related to the amount of lignin phenol present. Because of the COVID-19 outbreak, the importance of antiviral surfaces has been brought to light. Lignin is an interesting candidate for the development of antiviral coatings on a wide scale that are both economical and sustainable [74]. More research needs to be done on this substance. It is important to conduct research on innovative techniques, such as spray and brush coatings, which can be used to create resistant coatings for any kind of surface. In the future, it is recommended that the adhesive characteristics of the coating be investigated on a variety of substrates, including glass, wood, and plastic, among others [74].

**Table 4:** LigNPs as carriers of antibacterial agents.

Type of Lignin	NP Preparation Method	Loading Strategy	Particle Size (nm)	Antimicrobial Activity	References
Spherical					
Kraft, acetylated	Solvent displacement	Entrapment	160–1348	*S. aureus*, *S. epidermidis*, and *E. faecalis*	[75,76]
Alkali	Solvent displacement	Emulsion	~200	*Penicillium italicum*	[77]
Lignosulfonate, PNMA-modified	Self-assembling, chemical reduction	Adsorption	11	*S. aureus* and *E. coli*	[78]
Kraft	Solvent displacement	Ion exchange	60–200	*S. aureus*, *E. coli*, and *P. aeruginosa*	[79]
Alkali	Emulsion evaporation	Entrapment	117	*E. coli*	[80]
Quasi-spherical					
Lignosulfonate, modified with an azo dye	Chemical reduction to form ZnO	Coating	21–32	*S. haemolyticus*, *C. diphtheriae*, *B. cereus*, *R. ornithinolytica*, *S. typhimurium*, *S. paratyphi*, *A. fumigatus*, *A. penicilloides*, *C. albicans*, *C. coronatus*, and *M. cookei*	[81]
Organosolv	Chemical reduction to form AgNPs	Adsorption	28–54	*E. coli*	[82]
Irregular					
Kraft	Coating of lignin on silica followed by chemical reduction to form AgNP	Adsorption	30–36	*B. subtilis*, *S. aureus*, *P. aeruginosa*, *E. coli* and *K. pneumoniae*	[83]
Kraft	Acid precipitation	Infusion	40–70	*S. aureus*, *S. epidermidis*, *E. coli*, and *P. aeruginosa*	[84]

## 11. Conclusions and Prospects

This Perspective covers the latest lignin valorization methods and possibilities for food packaging, antimicrobial, and agricultural uses. The biopolymer lignin has many potential uses; however, it may be difficult to employ due to several difficulties. Overall, lignin structural and compositional heterogeneity is the biggest concern. This variability depends on the plant source and extraction method and requires a thorough study of the starting reagents and final products. Most applications of this biopolymer require a structure–activity dependence due to the wide variety of lignin variants. Lignin can be used as a green addition in food packaging to improve polymer-film mechanical and gas barrier qualities and give antioxidant and anti-UV action. Lignin’s incompatibility with the matrix complicates polymer film incorporation. To reduce heterogeneity and phase separation, lignin or nanoparticle functionalization must be adjusted to promote polymer film compatibility and dispersion. Understanding the relationship between lignin and packaged goods and film digestibility is also important. Lignin is not biodegradable under composting circumstances; hence, it is necessary to assess how lignin absorption affects product composability and biodegradability. The specific mechanism by which lignin interacts with bacteria, fungi, and viruses as an antibacterial agent is unknown. Its molecular weight, impurities, and reactive group content make lignin useful in medicine and biology, but also make it harder to verify its safety for human consumption.

The antibacterial activity of lignin in solution has been studied; however, few studies have used it as a coating material to construct antimicrobial surfaces. Since viruses and bacteria propagate through contact with contaminated surfaces and systematic disinfection is laborious, coatings that instantly inactivate germs are advantageous. Lignin coatings are easy to make and evaluate for antibacterial, antifungal, and antiviral properties. These coatings’ manufacturing and pathogen-fighting efficiency should be studied further. In the preceding portion of this Perspective, we discussed lignin’s agricultural uses, including fertilizer and processing. The current fertilizer application method is ineffective. Lignin is a good starting material for controlled-release fertilizers, but considerable drawbacks must be solved before their widespread use. Lignin-based fertilizer manufacturing needs improvement. Ammoxidation and Mannich processes add nitrogen to lignin, but the optimum reagents, byproducts, and working conditions can extend their lifespan. The same approach applies to using lignin as a covering material for controlled-release fertilizers or key element depots. Alternatively, lignin can be used to make novel fertilizers. Lignin nanoparticles and lignin modified with nutrients other than nitrogen, such as phosphorus, are exciting possibilities that should be researched. Lignin-based nanofertilizers present another opportunity to boost agricultural output. System development that releases nutrients near the plant, preventing nutrient waste and environmental pollution, seems promising. This Perspective showed how lignin, a natural chemical underutilized, has great promise for food packaging, antibacterial, agricultural, and other technological concerns that require ecologically acceptable solutions.

## Figures and Tables

**Table 1:** Overview of technical lignin extraction processes.

Process Type	Extraction Process Conditions		Solubility	Molecular Weight (kDa)	Poly-Dispersity	Impurities	Reference
	pH	Temperature					
Sulfur process (Sulfur lignin)							
Kraft lignin	NaOH, Na_2_S	170 °C	Alkali	0.1–3.0	2.5–3.5	Sulfur (1–3%) and ash (1–2%)	[20]
Lignosulfonates	SO_2_, Na^+^/Ca^+^/Mg^+^/NH_4_^+^	140 °C	Water	20–50	4.2–8.0	Sulfur (3.5–8%) and ash (4–8%)	[20]
Sulfur-free process (Non-Sulfur lignin)							
Organosolv lignin	acetic acid/formic acid/organic solvents	150–200 °C	Wide range of Organic solvents	0.5–4.0	1.3–4.0	Carbohydrates (1–3%) and ash (1.7%)	[21]
Soda lignin	NaOH	150–170 °C	Alkaline media pH > 10	0.8–3.0	2.5–3.5	Carbohydrates (1.5–3%) and ash (0.7–2.3%)	[21]

**Table 2:** Lignin as biodegradable polymer.

Lignin Type	Polymer Matrix	Tensile Strength (%)	Elongation at Break (%)	Gas Barrier Properties	Antioxidant Activity	UV-Barrier Activity	References
Soda lignin	PHB/PHA	85 ↑	77 ↓	Yes/Present	Yes/Present	Yes/Present	[24]
	Alginate	74 ↓	-	Yes/Present	Yes/Present	Yes/Present	[25]
	Chitosan	66 ↑	26 ↓	-	Yes/Present	-	[26]
	Poly(lactide)	27 ↓	43 ↓	-	Yes/Present	-	[27]
Kraft lignin	Starch	33 ↑	30 ↓	Yes/Present	-	-	[28]
	Agar	15 ↑	18 ↓	Yes/Present	-	Yes/Present	[29]
Organosolv lignin	Cellulose	-	-	-	Yes/Present	Yes/Present	[30]
Lignosulfonate	Poly(vinyl alcohol)	40 ↑	40 ↓	-	-	-	[31]

Polyhydroxybutyrate (PHB); polyhydroxyalkanoate (PHA); ↑ Higher (enhanced activity); ↓ reduced (decreased activity); - absent.
